# The added value of fasting blood glucose to serum squamous cell carcinoma antigen for predicting oncological outcomes in cervical cancer patients receiving neoadjuvant chemotherapy followed by radical hysterectomy

**DOI:** 10.1002/cam4.2414

**Published:** 2019-07-16

**Authors:** Miao‐Fang Wu, Mei‐Mei Guan, Chang‐Hao Liu, Jie‐Ying Wu, Qun‐Xian Rao, Jing Li

**Affiliations:** ^1^ Department of Gynecologic Oncology Sun Yat‐sen Memorial Hospital, Sun Yat‐sen University Guangzhou People's Republic of China

**Keywords:** cervical cancer, fasting blood glucose, neoadjuvant chemotherapy, prognosis, squamous cell carcinoma antigen

## Abstract

**Objective:**

To determine the combination of fasting blood glucose (FBG) with squamous cell carcinoma antigen (SCCA) assessments in the prediction of tumor responses to chemotherapy and pretreatment prognostication among patients receiving neoadjuvant chemotherapy (NACT) for locally advanced cervical cancer (LACC).

**Methods:**

Data of 347 LACC patients were retrospectively reviewed. Receiver operating characteristic (ROC) curves were constructed, and areas under the curves (AUCs) were compared to evaluate the ability to predict complete response (CR) following NACT. Patients were stratified into groups with low and high levels of SCCA and FBG and combined into low‐ or high‐SCCA and low‐ or high‐FBG groups. Cox regression analysis was performed to identify determinants of recurrence‐free survival (RFS) and overall survival (OS).

**Results:**

The AUCs were 0.70, 0.68, and 0.66 for SCCA, FBG, and a combination of SCCA and FBG for predicting CR following NACT, respectively; however, the differences among AUCs were not significant (*P* = .496). Pretreatment SCCA and FBG levels were identified as independent predictors of RFS and OS. The high‐SCCA/high‐FBG group showed significantly worse prognosis than the low‐SCCA/low‐FBG group. After adjusting for other variables, high‐SCCA/high‐FBG remained independently associated with an increased risk of tumor recurrence and death.

**Conclusion:**

SCCA, FBG, and a combination of SCCA and FBG could acceptably predict CR following NACT. Pretreatment SCCA and FBG levels were independent prognostic factors. The combination of SCCA and FBG levels refined the prognostic stratification of LACC patients, which allowed the group of patients with the highest risk of recurrence and death to be identified.

## 1 INTRODUCTION

Currently, over 85% of the global burden of cervical cancer is located in less developed countries.^1^ China bears a heavy burden of cervical cancer, with a high incidence of 7.5/100 000 and a mortality of 3.4/100 000.^2^ As a nation‐wide screening program has not been established in China, most new cases are diagnosed upon presentation at an advanced stage.^3^ Concurrent chemoradiotherapy (CCRT) is the current recommended standard treatment according to the National Comprehensive Cancer Network (NCCN) guidelines for patients with locally advanced disease (International Federation of Gynecology and Obstetrics [FIGO] stage IB2 and IIA2).^4^ However, impaired quality of life due to radiation‐induced ovarian failure is a significant outcome of CCRT which poses serious problems especially for young women.^5^ In addition, radiotherapy facilities are not always readily available to patients in developing countries. According to the NCCN Framework for Resource Stratification of NCCN Guideline, neoadjuvant chemotherapy (NACT) followed by radical surgery could be considered as an acceptable treatment for patients with locally advanced cervical cancer (LACC) who are from under‐developed regions.^6^ Because complete response (CR) following NACT is associated with significant long‐term survival benefits, it is considered a reliable surrogate endpoint of survival for LACC patients.^7^, ^8^ Given this, accurate assessment of the tumor response to NACT is critical to identify patients who will benefit the most from NACT and to predict prognosis.

Squamous cell carcinoma antigen (SCCA) has been identified as a predictive and prognostic factor for cervical cancer patients.^9^, ^10^, ^11^ Furthermore, the level of SCCA prior to NACT is reported to be an independent indicator of the chemotherapeutic response.^12^, ^13^ However, even in patients with equivalent pretreatment SCCA levels, LACC remains a biologically heterogeneous disease. Therefore, it is necessary to identify additional markers that could complement SCCA. There is a growing body of evidence that cancer patients with hyperglycemia have poor responses to chemotherapy.^14^, ^15^, ^16^, ^17^, ^18^ For cervical cancer patients, previous studies have revealed that an elevated level of fasting blood glucose (FBG) is a negative prognostic factor.^15^, ^18^, ^19^, ^20^ For LACC patients, we previously reported that hyperglycemia before NACT is independently associated with a decreased likelihood of CR.^15^ However, no data have supported that combining pretreatment SCCA and FBG levels improves the prediction of CR following NACT or refines the prognostic stratification of LACC patients. Therefore, we designed a retrospective cohort study to investigate the complementary role of FBG to SCCA in cervical cancer patients receiving NACT and radical hysterectomy for locally advanced disease.

## 2 MATERIALS AND METHODS

### 2.1 Patients

The medical records of cervical cancer patients who were treated at Sun Yat‐sen Memorial Hospital and the People's Hospital of Shaolin District between January 1, 2002, and January 1, 2012, were identified. The inclusion criteria were as follows: patients with FIGO stage IB2 and IIA2 disease, patients with histologically confirmed squamous cell carcinoma, adenocarcinoma, and adenosquamous carcinoma, patients with SCCA levels measured prior to NACT and patients who provided signed informed consent. The exclusion criteria were as follows: patients receiving any treatment at other institutions, patients with a history of previous chemotherapy or radiation therapy, and patients with a history of other types of malignancies. This study was approved by the Ethics Committee of Sun Yat‐sen Memorial Hospital and the People's Hospital of Shaolin District (Approval # SYSEC‐KY‐KS‐2019‐012).

Before NACT, all patients underwent gynecological examinations by at least two senior gynecologists. Blood samples were collected for laboratory tests within 1 week before initiation of NACT, and fasting was defined as no caloric intake for at least 8 hours. SCCA was assessed with an immunoradiometric assay kit (Imx, Abbott Diagnostics). FBG was measured using a glucose oxidase assay (Tosoh Corp., Tosoh, Japan).

The NACT regimens were as follows: TP, paclitaxel + cisplatin; FP, 5‐fluouracil + cisplatin; TC, paclitaxel + carboplatin; and BVP, bleomycin + vincristine +cisplatin. All patients received two to three cycles of NACT, and the cycles of NACT were based on the physician's judgment. Type III radical hysterectomy with pelvic lymphadenectomy was performed within 4 weeks after the last cycle of NACT. Pathological responses were retrospectively evaluated by at least two authorized pathologists. CR was defined as no evidence of viable tumor cells on the tumorous area.^21^ Postsurgical adjuvant radiotherapy was prescribed according to the NCCN guidelines.^4^

After the completion of therapy, all patients were followed‐up at 3‐month intervals for the first 2 years, every 6 months for the subsequent 3 years and annually thereafter. Each visit entailed a complete history and physical examination and a Papanicolaou smear of the vaginal vault. Follow‐up information was obtained via office visits or telephone interviews. When recurrence was suspected based on clinical findings, imaging studies or biopsies of suspicious lesions were performed on a case by‐case basis. Recurrence‐free survival (RFS) was calculated from the date of NACT until the date of the first relapse at any site. Overall survival (OS) was calculated from the date of NACT until the date of death due to any cause. Patients surviving past the last day of the follow‐up period were censored.

### 2.2 Statistical analysis

Statistical analyses were performed using STATA/SE (version 12.0, Stata Corp) and MedCalc (version 12.3.0, MedCalc Software). Receiver operating characteristic (ROC) curves were constructed to examine the predictive value of FBG and SCCA for CR following NACT. Optimal cutoff values were calculated by the maximum Youden indices. The areas under the curves (AUCs) were compared according to the method of DeLong et al^22^ RFS and OS were calculated with the use of the Kaplan‐Meier method and compared by the log‐rank test. For multiple comparisons of survival curves, the Bonferroni adjustment was applied. Cox proportional hazard models (enter method) were utilized to assess the impact of possible prognostic factors on patient survival. Hazard ratios (HRs) and 95% confidence intervals (95% CIs) were estimated. Factors with *P *＜ .15 in the univariate analysis were entered into a multivariate Cox regression model. All statistical tests were two‐sided, and a *P* < .05 was considered to be statistically significant.

## 3 RESULTS

### 3.1 Patient characteristics, the cutoff values of SCCA and FBG, and the complementary value of FBG to SCCA for predicting CR following NACT

A total of 347 patients met the study criteria. Their demographic profiles are summarized in Table 1. The median levels of SCCA and FBG were 5.6 ng/ml (range: 0.4‐15.6) and 5.1 mmol/l (range: 4.4‐10.1), respectively.

**Table 1:** Baseline characteristics

Characteristic	Overall (n = 347)	SCCA ≥ 6.2 ng/ml (n = 157)	SCCA < 6.2 ng/ml (n = 190)	*P* value	FBG ≥ 5.1 mmol/l (n = 193)	FBG < 5.1 mmol/l (n = 154)	*P* value
Age (y), median (range)	52 (24‐80)	51 (24‐80)	52 (26‐72)	.451	51 (26‐72)	52 (24‐80)	.813
BMI (kg/m^2^)	23.2 (19.4‐28.5)	23.1 (19.4‐28.5)	23.4 (20.9‐27.4)	.042	23.2 (19.4‐28.5)	23.2 (20.4‐28.4)	.443
FIGO Stage, n (%)							
IB2	176 (50.7)	75 (47.8)	101 (53.2)	.318	99 (51.3)	77 (50.0)	.810
IIA2	171 (49.3)	82 (52.2)	89 (46.8)		94 (48.7)	77 (50.0)	
CR achieved, n (%)							
No	260 (74.9)	141 (89.8)	119 (62.6)	<.0001	166 (86.0)	94 (61.0)	<.0001
Yes	87 (25.1)	16 (10.2)	71 (37.4)		27 (14.0)	60 (39.0)	
NACT regimen, n (%)							
Cisplatin + paclitaxel	307 (88.5)	140 (89.2)	167 (87.9)	.500	169 (87.6)	138 (89.6)	.072
Cisplatin‐based	40 (11.5)	17 (10.8)	23 (12.1)		24 (12.4)	16 (10.4)	
Tumor histology, n (%)							
SCC	289 (83.3)	135 (86.0)	154 (81.1)	.220	163 (84.5)	126 (81.8)	.513
NSCC	58 (16.7)	22 (14.0)	36 (19.0)		30 (15.5)	28 (18.2)	
Differentiation, n (%)							
Grade 1‐2	302 (87.0)	136 (86.6)	166 (87.4)	.837	168 (87.1)	134 (87.0)	.993
Grade 3	45 (13.0)	21 (13.4)	24 (12.6)		25 (13.0)	20 (13.0)	
Deep stromal invasion, n (%)							
No	61 (17.6)	24 (15.3)	37 (19.5)	.308	35 (18.1)	26 (16.9)	.761
Yes	286 (82.4)	133 (84.7)	153 (80.5)		158 (81.9)	128 (83.1)	
LVSI, n (%)							
No	146 (42.1)	61(38.9)	85 (44.7)	.269	86 (44.6)	60 (39.0)	.294
Yes	201 (57.9)	96 (61.2)	105 (55.3)		107 (55.4)	94 (61.0)	
Positive surgical margin, n (%)							
No	333 (96.0)	151 (96.2)	182 (95.8)	.855	182 (94.3)	151 (98.1)	.078
Yes	14 (4.0)	6 (3.8)	8 (4.2)		11 (5.7)	3 (2.0)	
Positive nodes, n (%)							
No	211 (60.8)	81 (51.6)	130 (68.4)	.001	112 (58.0)	99 (64.3)	.236
Yes	136 (39.2)	76 (48.4)	60 (31.6)		81 (42.0)	55 (35.7)	
Positive parametrium, n (%)							
No	330 (95.1)	145 (92.4)	185 (97.4)	.031	182 (94.3)	148 (96.1)	.439
Yes	17 (4.9)	12 (7.6)	5 (2.6)		11 (5.7)	6 (3.9)	
Postsurgical CCRT, n (%)							
No	53 (15.3)	14 (8.9)	39 (20.5)	.003	26 (13.5)	27 (17.5)	.296
Yes	294 (84.7)	143 (91.1)	151 (79.5)		167 (86.5)	127 (82.5)	

Abbreviations: BMI, body mass index; CCRT, concurrent chemoradiothrapy; CR, complete response; FBG, fasting blood glucose; FIGO, International Federation of Gynecology and Obstetrics; LVSI, lymphatic vascular space involvement; NACT, neoadjuvant chemotherapy; NSCC, nonsquamous cell carcinoma; SCC, squamous cell carcinoma; SCCA, squamous cell carcinoma antigen.

**Figure 1:**
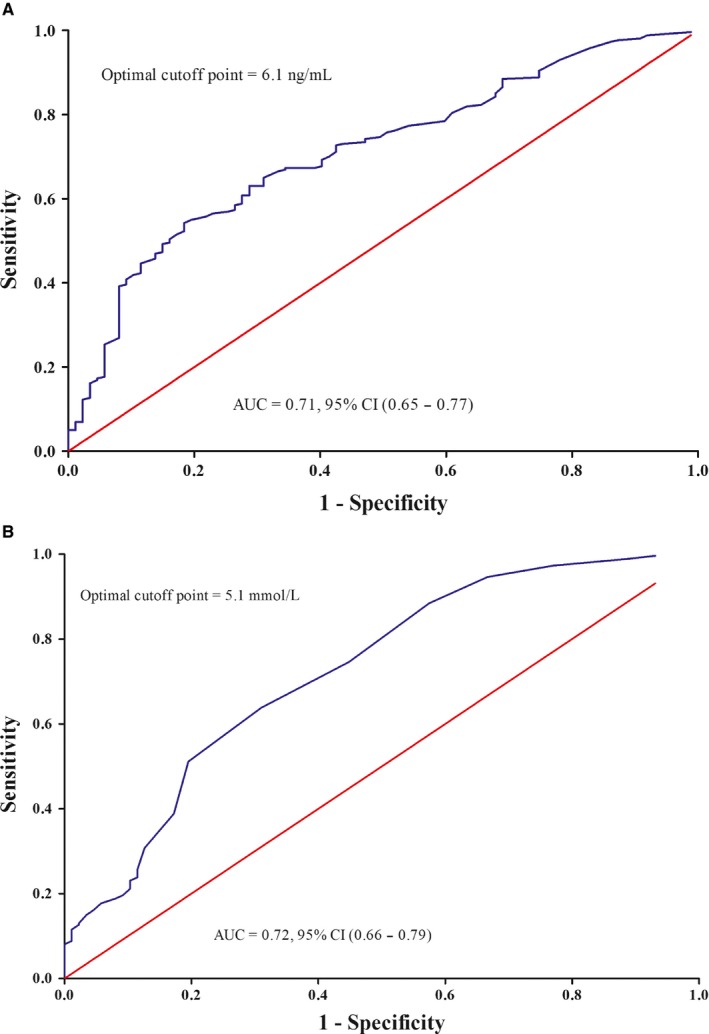
Receiver operating characteristic curve (ROC) analysis of squamous cell carcinoma antigen (SCCA) and fasting blood glucose (FBG) for the prediction of complete response following neoadjuvant chemotherapy. A. SCCA. B. FBG

ROC curves were generated, and the AUCs for predicting CR following NACT were 0.71 (95% CI 0.65‐0.77, *P* < .0001) and 0.72 (95% CI 0.66‐0.79, *P* < .0001) for SCCA (Figure 1A) and FBG (Figure 1B), respectively. SCCA levels ≥ 6.2 ng/ml yielded the maximum Youden's index with 54.23% (95% CI 0.48‐0.60) sensitivity and 81.61% (95% CI 0.72‐0.89) specificity. FBG ≥ 5.1 mmol/l yielded the maximum Youden's index with 63.85% (95% CI 0.58‐0.70) sensitivity and 68.97% (95% CI 0.58‐0.78) specificity. Patient characteristics according to SCCA levels and FBG levels are summarized in Table 1. The high‐SCCA group had significantly more patients with higher levels of body mass indexes (BMIs), lymph node metastasis, and positive parametrium and more patients who did not achieve CR following NACT and receiving CCRT. The high‐FBG group had significantly more women who did not achieve CR after NACT. In addition, time‐dependent ROC curve analyses were conducted to evaluate the prognostic value of SCCA and FBG. For the RFS prediction, the areas under the ROC curve at 12‐month, 24‐month, and 60‐month were 0.71, 0.63, and 0.68, respectively, in FBG and 0.66, 0.66, and 0.67 respectively, in SCCA. For the OS prediction, the areas under the ROC curve at 12‐month, 24‐month, and 60‐month were 0.90, 0.72, and 0.62 respectively, in FBG and 0.47, 0.60, and 0.67 respectively, in SCCA.

**Figure 2:**
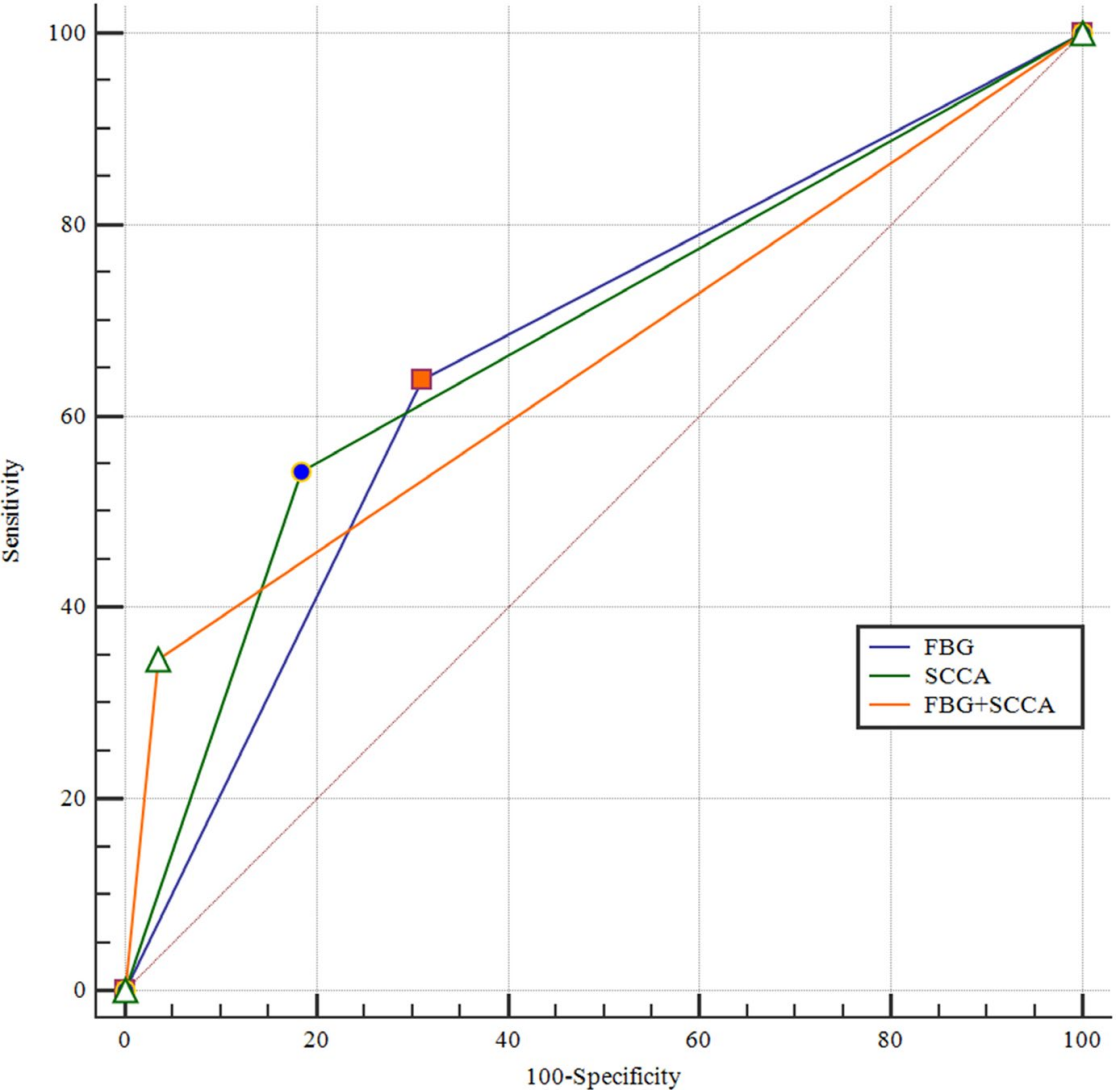
Receiver operating characteristic (ROC) curves of squamous cell carcinoma antigen (SCCA), fasting blood glucose (FBG), and the combination of the two individual markers for predicting complete response (CR) following neoadjuvant chemotherapy (NACT)

To assess the value of SCCA, FBG, and SCCA plus FBG for predicting CR following NACT, we perform a pairwise comparison of the AUCs (Figure 2); however, no significant difference was identified (total *P* = .496; AUC for SCCA: 0.70, 95% CI 0.61‐0.71, *P* < .0001; AUC for FBG: 0.68, 95% CI 0.63‐0.73, *P* < .0001; AUC for SCCA plus FBG: 0.66, 95% CI 0.60‐0.71, *P* < .0001).

### 3.2 Comparison of RFS and OS stratified by pretreatment SCCA and FBG

The median follow‐up time was 37 months (range: 4‐66). Figure S1 demonstrates the survival curves for RFS and OS. Recurrence and death were noted in 84 patients and 88 patients, respectively. The Kaplan‐Meier survival graphs showed a statistically significant difference in RFS between the groups categorized by SCCA (log‐rank test *P* < .0001) and FBG (log‐rank test *P* < .0001), respectively. Similarly, the difference in OS between the groups categorized by SCCA (log‐rank test *P* < .0001) and FBG (log‐rank test *P* < .0001) was statistically significant.

Table 2 summarizes the results of the univariate and multivariate Cox proportional hazard analyses. SCCA ≥ 6.2 ng/ml and FBG ≥ 5.1 mmol/l were independently associated with RFS (SCCA: adjusted HR 2.53, 95% CI 1.54‐4.15, *P* < .0001; FBG: adjusted HR 2.19, 95% CI 1.29‐3.73, *P* = .004) and OS (SCCA: adjusted HR 1.88, 95% CI 1.18‐2.99, *P* = .008; FBG: adjusted HR 1.93, 95% CI 1.16‐3.20, *P* = .011).

**Table 2:** Cox proportional hazards regression models of risk factors associated with survival outcomes

	Recurrence‐free survival						Overall survival					
	Univariate analysis			Multivariate analysis			Univariate analysis			Multivariate analysis		
	HR	95% CI	*P* value	HR	95% CI	*P* value	HR	95% CI	*P* value	HR	95% CI	*P* value
Age (y)	1.00	0.97‐1.02	.825				0.99	0.97‐1.01	.386			
BMI (kg/m^2^)	1.05	0.92‐1.20	.458				1.08	0.95‐1.23	.245			
FIGO Stage (IIA2 vs IB2)	1.18	0.95‐1.47	.132	1.00	0.79‐1.25	.976	1.12	0.91‐1.38	.299			
CR achieved (no vs yes)	8.02	2.94‐21.90	<.0001	2.52	0.87‐7.30	.088	11.00	3.48‐34.81	<.0001	4.22	1.28‐13.91	.018
NACT regimen (cisplatin + paclitaxel vs cisplatin‐based)	1.10	0.58‐2.07	.770				0.72	0.35‐1.50	.384			
Tumor histology (NSCC vs SCC)	1.62	0.97‐2.70	.064	1.41	0.82‐2.42	.216	1.50	0.90‐2.49	.119	1.25	0.74‐2.12	.404
Differentiation (Grade 3 vs Grade 1‐2)	0.91	0.47‐1.77	.785				0.89	0.46‐1.73	.740			
Deep stromal invasion (yes vs no)	2.03	1.01‐4.05	.045	1.24	0.60‐2.57	.556	2.21	1.11‐4.41	.024	1.33	0.65‐2.70	.437
LVSI (yes vs no)	1.24	0.80‐1.92	.345				1.21	0.79‐1.87	.381			
Positive surgical margin (yes vs no)	11.60	6.37‐21.10	<.0001	5.91	3.02‐11.56	<.0001	8.18	4.56‐14.66	<.0001	4.31	2.24‐8.30	<.0001
Positive nodes (yes vs no)	4.14	2.61‐6.57	<.0001	2.64	1.62‐4.28	<.0001	4.36	2.75‐6.91	<.0001	2.80	1.73‐4.53	<.0001
Positive parametrium (yes vs no)	8.69	4.83‐15.63	<.0001	4.12	2.19‐7.75	<.0001	6.65	3.64‐12.16	<.0001	2.92	1.54‐5.51	.001
SCCA (≥ 6.2 ng/ml vs < 6.2 ng/ml)	3.07	1.92‐4.90	<.0001	2.53	1.54‐4.15	<.0001	2.42	1.56‐3.76	<.0001	1.88	1.18‐2.99	.008
FBG (≥5.1 mmol/l vs < 5.1 mmol/l)	2.76	1.66‐4.60	<.0001	2.19	1.29‐3.73	.004	2.43	1.49‐3.97	<.0001	1.93	1.16‐3.20	.011

Abbreviations: BMI, body mass index; CI, confidence interval; CR, complete response; FBG, fasting blood glucose; FIGO, International Federation of Gynecology and Obstetrics; HR, hazard ratio; LVSI, lymphatic vascular space involvement; NACT, neoadjuvant chemotherapy; NSCC, nonsquamous cell carcinoma; SCC, squamous cell carcinoma; SCCA, squamous cell carcinoma antigen;

### 3.3 Prognostic significance of integrating SCCA and FBG

**Figure 3:**
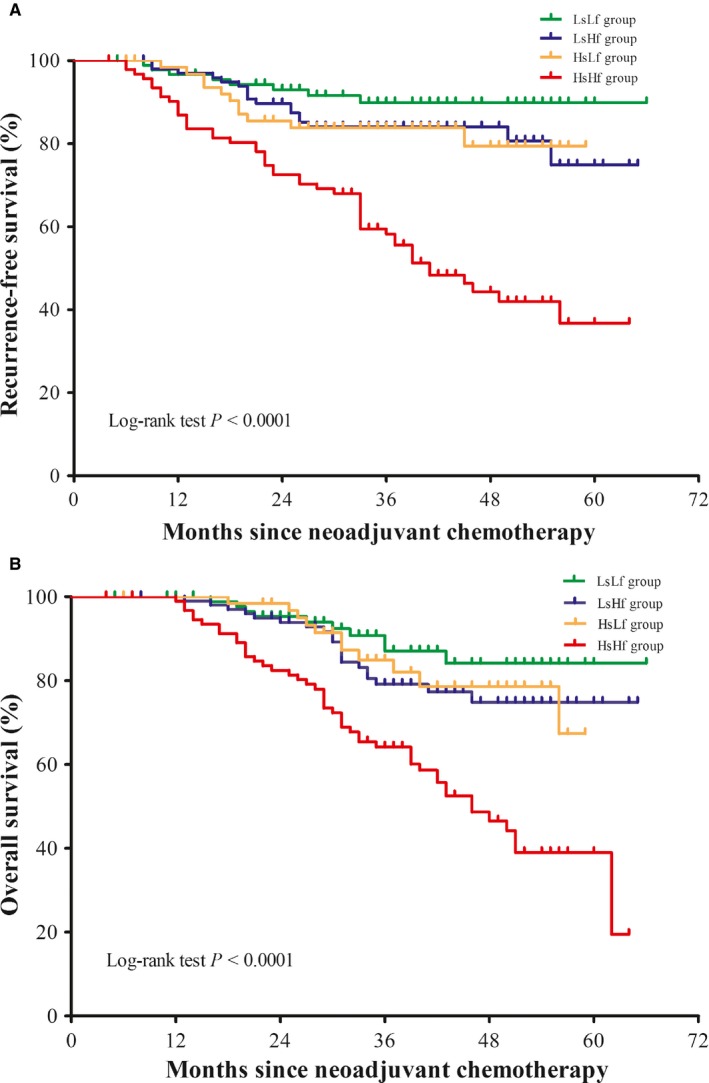
Kaplan‐Meier curves for recurrence‐free survival (RFS) and overall survival (OS). A. RFS (log‐rank test *P* < .0001). B. OS (log‐rank test *P* < .0001). LsLf group = patients with low SCCA and low FBG (SCCA < 6.2 ng/ml and FBG < 5.1 mmol/l), LsHf group = patients with low SCCA and high FBG (SCCA < 6.2 ng/ml and FBG ≥ 5.1 mmol/l), HsLf group = patients with high SCCA and low FBG (SCCA ≥ 6.2 ng/ml and FBG < 5.1 mmol/l), HsHf group = patients with high SCCA and high FBG (SCCA ≥ 6.2 ng/ml and FBG ≥ 5.1 mmol/l). FBG, fasting blood glucose. SCCA, squamous cell carcinoma antigen

Because elevated SCCA levels and FBG levels prior to NACT were independent prognosticators of RFS and OS, we performed a further investigation to evaluate a new molecular classification by integrating the two biomarkers to improve patient prognostic stratification. Accordingly, the study population was divided into four subgroups: low SCCA and low FBG [LsLf, SCCA < 6.2 ng/ml and FBG < 5.1 mmol/l; n = 90 (25.9%)], low SCCA and high FBG [LsHf, SCCA < 6.2 ng/ml and FBG ≥ 5.1 mmol/l; n = 100 (28.8%)], high SCCA and low FBG [HsLf, SCCA ≥ 6.2 ng/ml and FBG < 5.1 mmol/l; n = 64 (18.4%)], and high SCCA and high FBG [HsHf, SCCA ≥ 6.2 ng/ml and FBG ≥ 5.1 mmol/l; n = 93 (26.8%)]. Kaplan‐Meier curves for RFS and OS are displayed in Figure 3. The differences in RFS and OS among the four groups were significant (log‐rank test *P* < .0001). According to the post hoc Bonferroni analysis (Table S1), the differences in RFS between the HsHf group and the HsLf group (log‐rank test *P* < .0001), the HsHf group and the LsHf group (log‐rank test *P* < .0001), and the HsHf group and the LsLf group (log‐rank test *P* < .0001) were statistically significant. Similarly, a post hoc Bonferroni analysis (Table S2) identified significant differences in OS between the HsHf group and the HsLf group (log‐rank test *P* = .001), the HsHf group and the LsHf group (log‐rank test *P* < .0001), and the HsHf group and the LsLf group (log‐rank test *P* < .0001).

Using the LsLf group as a reference (Table 3), the unadjusted HRs for RFS and OS of the HsHf group were 6.28 (95% CI 2.97‐13.28, *P* < .0001) and 4.49 (95% CI 2.27‐8.88, *P* < .0001), respectively. After adjusting for other prognostic factors, the HRs for the RFS and OS in the HsHf group were 3.91 (95% CI 1.79‐8.54, *P* = .001) and 2.60 (95% CI 1.29‐5.23, *P = *.008).

**Table 3:** Prognostic value of combination of SCCA and FBG for recurrence‐free survival and overall survival

	Unadjusted HR	95% CI	*P* value	Adjusted HR^a^	95% CI	*P* value
Recurrence‐free survival						
SCCA < 6.2 ng/ml + FBG <5.1 mmol/l (LsLf group)	Reference			Reference		
SCCA < 6.2 ng/ml + FBG ≥5.1 mmol/l (LsHf group)	1.87	0.81‐4.33	.145	1.25	0.52‐2.99	.622
SCCA ≥ 6.2 ng/ml + FBG <5.1 mmol/l (HsLf group)	2.00	0.80‐4.97	.137	1.36	0.54‐3.43	.515
SCCA ≥ 6.2 ng/ml + FBG ≥5.1 mmol/l (HsHf group)	6.28	2.97‐13.28	<.0001	3.91	1.79‐8.54	.001
Overall survival						
SCCA < 6.2 ng/ml + FBG <5.1 mmol/l (LsLf group)	Reference			Reference		
SCCA < 6.2 ng/ml + FBG ≥5.1 mmol/l (LsHf group)	1.66	0.78‐3.55	.190	1.08	0.49‐2.38	.841
SCCA ≥ 6.2 ng/ml + FBG <5.1 mmol/l (HsLf group)	1.54	0.65‐3.62	.325	0.96	0.40‐2.29	.929
SCCA ≥ 6.2 ng/ml + FBG ≥5.1 mmol/l (HsHf group)	4.49	2.27‐8.88	<.0001	2.60	1.29‐5.23	.008

Abbreviations: CI, confidence interval; FBG, fasting blood glucose; HR, hazard ratio; SCCA, squamous cell carcinoma antigen.

a Adjusted HRs for recurrence‐free survival were adjusted for International Federation of Gynecology and Obstetrics stage (IIA2 vs IB2), complete response (yes vs no), tumor histology (nonsquamous cell carcinoma vs squamous cell carcinoma), deep stromal invasion (yes vs no), positive surgical margin (yes vs no), positive nodes (yes vs no), and positive parametrium (yes vs no), adjusted HRs for overall survival were adjusted for complete response (yes vs no), tumor histology (nonsquamous cell carcinoma vs squamous cell carcinoma), deep stromal invasion (yes vs no), positive surgical margin (yes vs no), positive nodes (yes vs no), and positive parametrium (yes vs no).

## 4 DISCUSSION

NACT is an alternative treatment particularly for LACC patients in areas where radiotherapy facilities are scarce.^6^ Many Chinese institutions have used NACT for many years as a common strategy for patients with FIGO stage IB2 and IIA2 disease.^23^ Because the optimal pathological response has been validated as a strong predictor of survival, CR following NACT is utilized as a reliable surrogate endpoint of survival for patients receiving NACT for LACC.^7^, ^8^ The prognostic value of CR was also confirmed in our patient cohort. Given current evidence, we believe that improvements in the pretreatment prediction of patient responses to NACT and further prognostic discrimination are significant priorities.

To our knowledge, this is the first study to assess the complementary role of FBG to SCCA for predicting tumor responses to NACT and prognostic stratification among LACC patients. We found that elevated levels of SCCA and FBG prior to NACT were independent predictors for decreased RFS and OS. Furthermore, the presence of high levels of SCCA and FBG was independently associated with an approximately threefold increase in the risk of tumor recurrence and death compared with low levels of SCCA and FBG. In addition, in the ROC curve analysis, SCCA, FBG, and the combination of the two individual markers had acceptable predictive capabilities of CR following NACT; however, we did not find that FBG provided complementary predictive value to SCCA.

As a subfraction of the tumor‐associated antigen,^24^ SCCA has been reported as a prognostic marker in cervical cancer patients. For LACC patients, the present study confirmed previous findings, which showed that pretreatment SCCA levels are independently associated with patient prognosis.^9^, ^10^, ^11^ In addition, there is a growing body of evidence that an increased level of SCCA is an indicator of poor response to chemotherapy for cervical cancer patients.^12^, ^13^ Using immunohistochemistry analysis, Chen et al reported that SCCA expression levels in tumor tissues are a predictive indicator of chemosensitivity of LACC patients who are treated by NACT.^25^ Our previous study and the study by Li et al showed that LACC patients with an elevated level of pretreatment SCCA in the serum are more likely to have a poor response to NACT.^12^, ^15^ In the current study, we further assessed the predictive value of SCCA and found that the baseline SCCA level could be used as a moderate predictor of CR following NACT (AUC = 0.70, 95% CI 0.61‐0.71, *P* < .0001).

The prognostic significance of hyperglycemia in cervical cancer patients with advanced disease has been reported in the literature.^18^ We have also previously reported that LACC patients with hyperglycemia prior to NACT have a decreased likelihood of achieving CR compared with those with euglycemia.^15^ These findings were confirmed by the current study. Furthermore, we reported here that pretreatment FBG levels had an acceptable ability to predict CR following NACT (AUC = 0.68, 95% CI 0.63‐0.73, *P* < .0001). Possible explanations for the negative impact of hyperglycemia on cancer treatment outcomes are as follows. First, hyperglycemia provides a high glucose fuel source that helps cancer cells maintain rapid proliferation.^26^ Second, up‐regulated expression of vascular endothelial growth factor (VEGF) can be induced in the hyperglycemic environment, which is a marker of enhanced tumor aggressiveness.^27^, ^28^ Third, hyperinsulinemia is another consequence of hyperglycemia, which can stimulate cell proliferation by activating insulin‐like growth factor‐I (IGF‐I).^29^, ^30^ Fourth, high levels of blood glucose may cause inflammation, which can result in the release of cytokines that can enhance cancer growth.^28^

Given the significance of SCCA and FBG for LACC patients, it is reasonable to combine these biomarkers, and this combination was expected to provide more useful information for both physicians and patients. As hypothesized, we found that combining SCCA and FBG refined the prognostic stratification of LACC patients. This combination led to the identification of 26.8% LACC patients as being at the highest risk of progression. Of the 93 patients who were reclassified as highest risk in our study, 84 (90.3%) received CCRT. However, their prognosis remained ominous. Therefore, further studies are required to evaluate whether these patients could gain a survival benefit from more intensive comprehensive management. In addition, our results suggested that the combination of SCCA and FBG had an acceptable ability to predict CR following NACT. SCCA is reported to inhibit the activity of serine protease and cysteine proteinase.^31^, ^32^ The precise mechanism by which the combination of SCCA and FBG could improve the prognostic stratification of LACC patients is less clear. SCCA and glucose involve different signaling pathways, which may be a possible explanation. On the other hand, with regard to the ability to predict CR following NACT, the present study did not find that the combined magnitude was superior to the individual effect of either marker alone. Considering our relatively small sample size, we believe that studies with a larger number of patients are needed to further explore the complementary role of evaluating FBG in addition to SCCA in such cases.

There are several limitations to this study. First, unbalanced and unrecognized bias may be present due to its retrospective nature. Second, the level of FBG can be influenced by many factors including antidiabetic drugs. However, these factors were not documented in every patient and serial dynamic serum levels of FBG was lacking. Accordingly, the potential influence from these factors could not be eliminated. Third, our study did not explore whether the duration of hyperglycemia could influence treatment outcomes. Fourth, our data were obtained only from Chinese patients, and the results were not validated using an external dataset. Despite these limitations, the long‐term follow‐up time of our study enabled us to identify most cases of relapse because the majority of recurrences among cervical cancer patients are detected within 2 years of primary treatment.^33^ Additionally, our work is the first to show the novel use of FBG and SCCA, not only in predicting tumor response to chemotherapy but also as a novel prognostic classification factor for LACC patients treated with NACT and RH.

In conclusion, our data suggest that pretreatment FBG adds prognostic value to SCCA. Therefore, FBG can be utilized as a prognosis stratification marker together with SCCA in LACC patients. In addition, SCCA, FBG, and the combination of the two markers can acceptably predict tumor responses to NACT. Because blood glucose is an inexpensive and easily measurable marker in clinical practice, the use of FBG in combination with SCCA may have important implications.

## CONFLICT OF INTEREST

The authors declare that they have no conflict of interest.

## Data Availability

The data that support the findings of this study are available on request from the corresponding author.
