# Supplementary figures and images for: The added value of fasting blood glucose to serum squamous cell carcinoma antigen for predicting oncological outcomes in cervical cancer patients receiving neoadjuvant chemotherapy followed by radical hysterectomy
Source: Cancer Med. 2019 Jul 16;8(11):5068–78. doi: 10.1002/cam4.2414 (PMC6718550; doi:10.1002/cam4.2414)

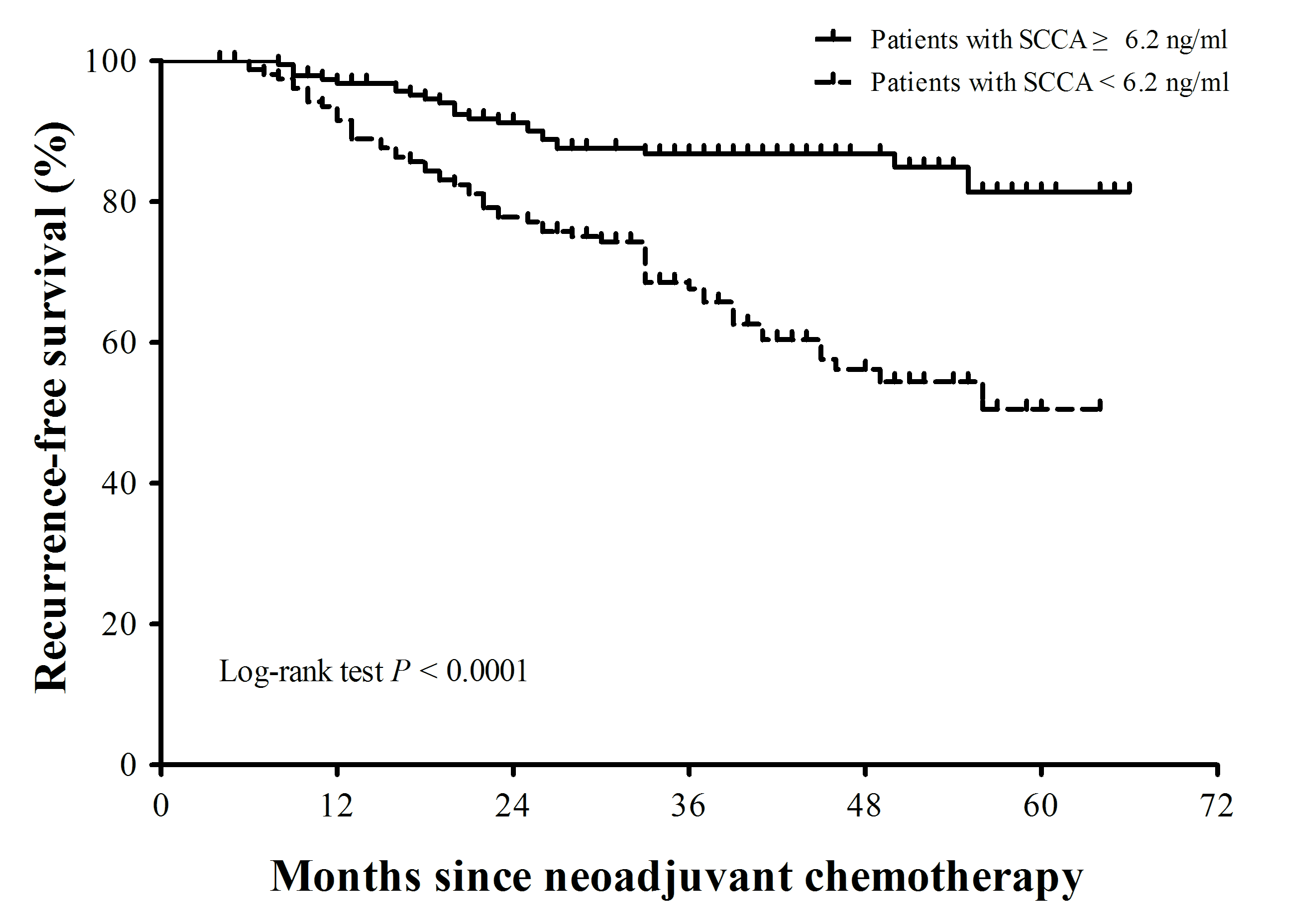

Supplement: Supplementary file 1 [file CAM4-8-5068-s001.tif]

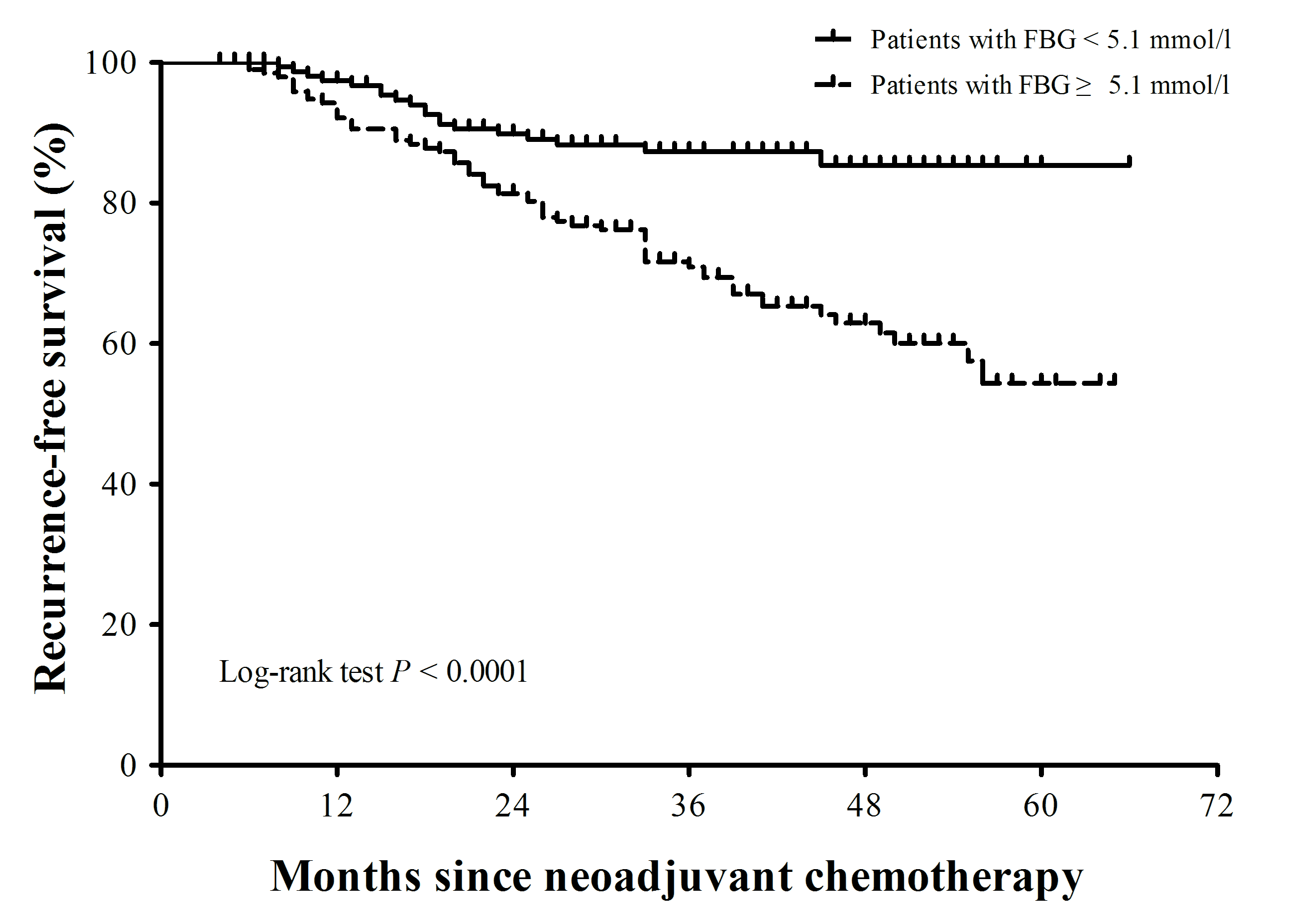

Supplement: Supplementary file 2 [file CAM4-8-5068-s002.tif]

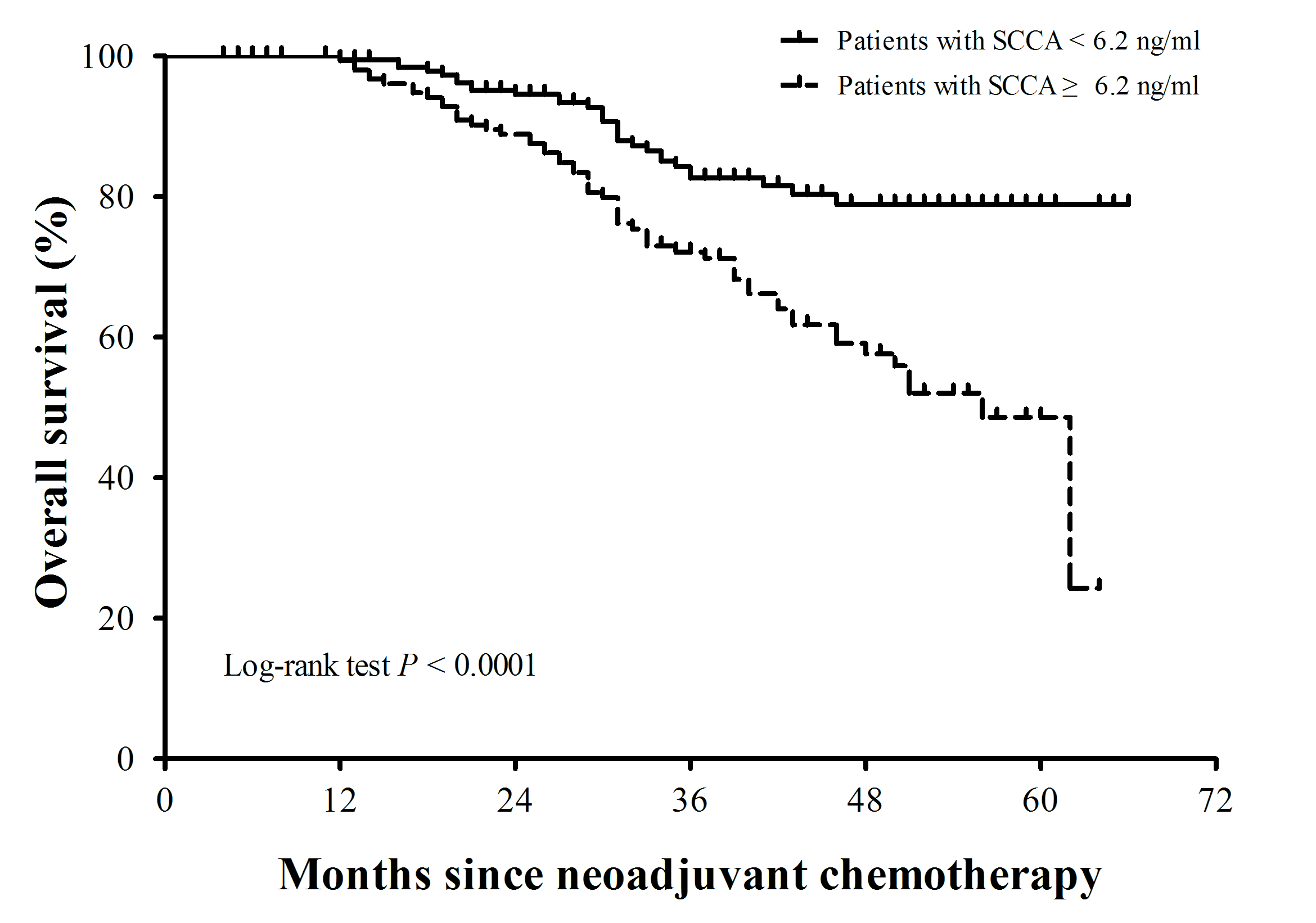

Supplement: Supplementary file 3 [file CAM4-8-5068-s003.tif]

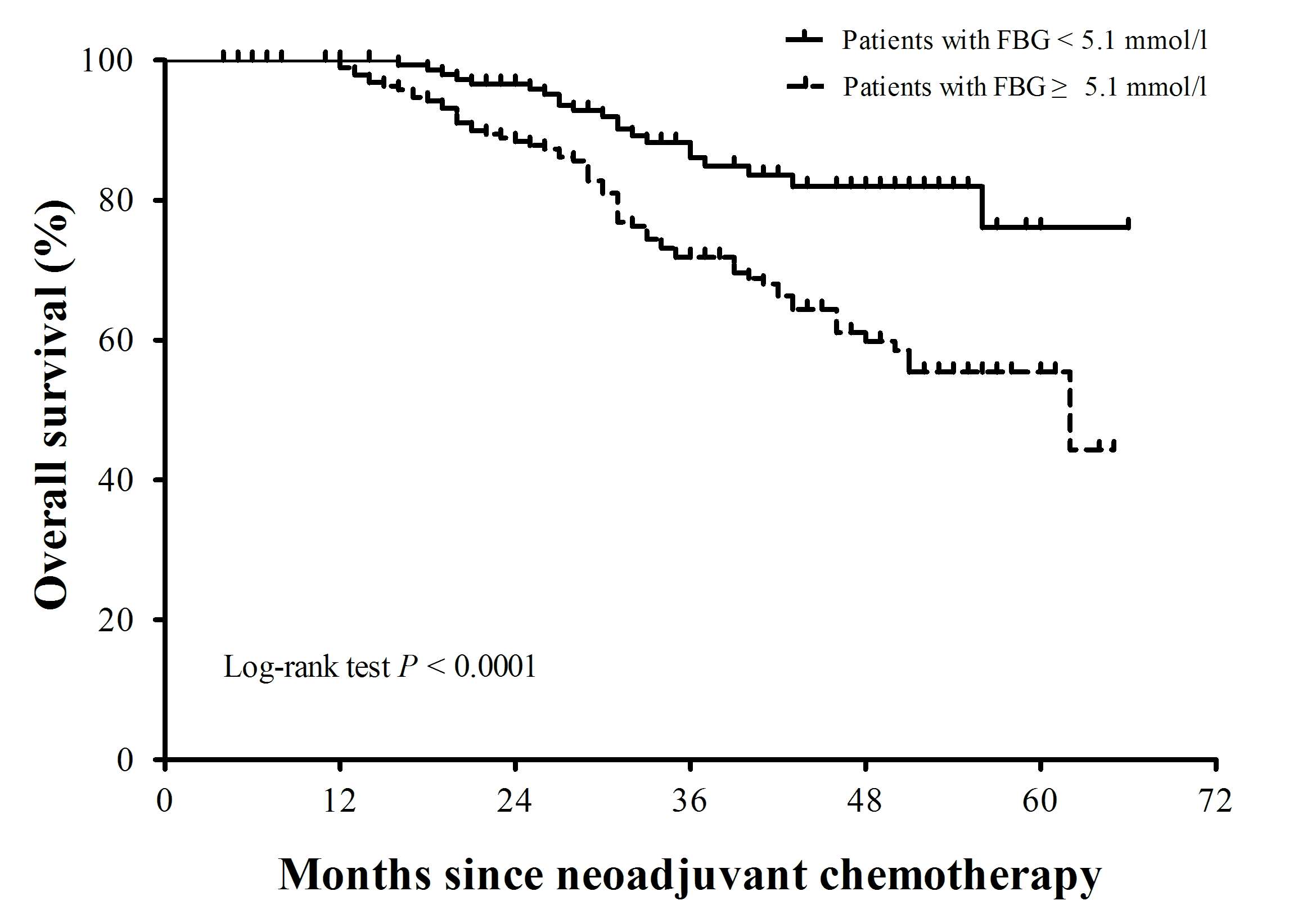

Supplement: Supplementary file 4 [file CAM4-8-5068-s004.tif]
