# Supplementary material for: The added value of fasting blood glucose to serum squamous cell carcinoma antigen for predicting oncological outcomes in cervical cancer patients receiving neoadjuvant chemotherapy followed by radical hysterectomy
Source: Cancer Med. 2019 Jul 16;8(11):5068–78. doi: 10.1002/cam4.2414 (PMC6718550; doi:10.1002/cam4.2414)
Supplement: Supplementary file 5 [file CAM4-8-5068-s005.doc]

Supplementary table 1. Comparison of recurrence-free survival using log-rank test with Bonferroni correction

|  | LsLf group | | LsHf group | | HsLf group | | HsHf group | |
| --- | --- | --- | --- | --- | --- | --- | --- | --- |
|  | Chi-Square | *P* value | Chi-Square | *P* value | Chi-Square | *P* value | Chi-Square | *P* value |
| LsLf group | — | — | 2.22 | 0.136 | 2.12 | 0.145 | 30.40 | <0.0001 |
| LsHf group | 2.22 | 0.136 | — | — | 0.02 | 0.890 | 20.98 | <0.0001 |
| HsLf group | 2.12 | 0.145 | 0.02 | 0.890 | — | — | 13.04 | <0.0001 |
| HsHf group | 30.40 | <0.0001 | 20.98 | <0.0001 | 13.04 | <0.0001 | — | — |

LsLf group: SCCA< 6.2 ng/ml + FBG < 5.1 mmol/l;

LsHf group: SCCA< 6.2 ng/ml + FBG ≥ 5.1 mmol/l;

HsLf group: SCCA≥ 6.2 ng/ml + FBG < 5.1 mmol/l;

HsHf group: SCCA≥ 6.2 ng/ml + FBG ≥ 5.1 mmol/l;
