# Supplementary material for: The added value of fasting blood glucose to serum squamous cell carcinoma antigen for predicting oncological outcomes in cervical cancer patients receiving neoadjuvant chemotherapy followed by radical hysterectomy
Source: Cancer Med. 2019 Jul 16;8(11):5068–78. doi: 10.1002/cam4.2414 (PMC6718550; doi:10.1002/cam4.2414)
Supplement: Supplementary file 6 [file CAM4-8-5068-s006.doc]

Supplementary table 2. Comparison of overall survival using log-rank test with Bonferroni correction

|  | LsLf group | | LsHf group | | HsLf group | | HsHf group | |
| --- | --- | --- | --- | --- | --- | --- | --- | --- |
|  | Chi-Square | *P* value | Chi-Square | *P* value | Chi-Square | *P* value | Chi-Square | *P* value |
| LsLf group | — | — | 1.87 | 0.172 | 0.91 | 0.341 | 22.49 | 0.000 |
| LsHf group | 1.87 | 0.172 | — | — | 0.06 | 0.809 | 15.22 | 0.000 |
| HsLf group | 0.91 | 0.341 | 0.06 | 0.809 | — | — | 11.11 | 0.001 |
| HsHf group | 22.49 | 0.000 | 15.22 | 0.000 | 11.11 | 0.001 | — | — |

LsLf group: SCCA< 6.2 ng/ml + FBG < 5.1 mmol/l;

LsHf group: SCCA< 6.2 ng/ml + FBG ≥ 5.1 mmol/l;

HsLf group: SCCA≥ 6.2 ng/ml + FBG < 5.1 mmol/l;

HsHf group: SCCA≥ 6.2 ng/ml + FBG ≥ 5.1 mmol/l;
