# Supplementary material for: The added value of fasting blood glucose to serum squamous cell carcinoma antigen for predicting oncological outcomes in cervical cancer patients receiving neoadjuvant chemotherapy followed by radical hysterectomy
Source: Cancer Med. 2019 Jul 16;8(11):5068–78. doi: 10.1002/cam4.2414 (PMC6718550; doi:10.1002/cam4.2414)
Supplement: Supplementary file 7 [file CAM4-8-5068-s007.docx]

Supplementary Figure 1. Kaplan-Meier curves for recurrence-free survival (RFS) and overall survival (OS). A. RFS by squamous cell carcinoma antigen (SCCA) group (*P* < 0.0001). B. RFS by FBG group (*P* < 0.0001). C. OS by SCCA group (*P* < 0.0001). D. OS by FBG group (*P* < 0.0001). Footnote: FBG, fasting blood glucose. SCCA, squamous cell carcinoma antigen.
